# Determination and managerial implications of machine conditions for high-grade industrial polycaprolactam (nylon 6)

**DOI:** 10.1038/s41598-023-37383-8

**Published:** 2023-07-04

**Authors:** Moses Olabhele Esangbedo, Johnson Kehinde Abifarin

**Affiliations:** 1 School of Management Engineering, Xuzhou University of Technology, Xuzhou, China; 2 School of Engineering, College of Engineering and Computer Science, Australian National University, Australian Capital Territory, Canberra, 2601 Australia

**Keywords:** Mechanical engineering, Structural materials, Mathematics and computing

## Abstract

Polycaprolactam (PA6) is a thermoplastic polymeric material and because of its excellent mechanical properties, it has found an extensive application in military, textile, biomedical, building and construction, and several others. Because of its extensive applications, machine turning operation becomes a crucial section in the manufacturing of high-grade PA6. Hence, to have a high-grade PA6, turning operational conditions (cutting speed, feed rate and depth of cut) are optimized on the three surface profile responses and one material removal rate (MMR) with help probability based multi-response optimization analysis. This analysis is employed for an efficient multi-criterial decision making when PA6 is manufactured with a turning operation machine. The result revealed an optimal turning operational conditions to be 860 rpm cutting speed, 0.083 mm/rev feed rate, and 4 mm depth of cut. Furthermore, the analysis of variance and the numerical presentation of the turning operational conditions revealed that the feed rate is the most significant condition with a contribution of 34.09%, followed by cutting speed with a contribution of 32.05%, and then depth of cut with a contribution of 28.62%. Also, the confirmation analysis revealed a very high efficacy of the multi-objective optimization method employed in this study. This suggests that probability based multi-objective optimization is efficacious for optimizing machine conditions of any manufactured engineering material. It is interesting to state that the high confidence level placed on the considered turning operational conditions gives room for probable machine conditions adjustments for better PA6 in the case where different machine types are employed.

## Introduction

Nylon is a synthetic thermoplastic polymer frequently used in injection moulding applications; it is sometimes known as Polycaprolactam (P) informally. There are two types of nylon injection moulding: PA6 (nylon 6) and PA66 (nylon 66)^1,2^. The physical and chemical characteristics of PA66 and PA6 are strikingly similar. PA6 is more soluble and impact resistant than PA66, but it also absorbs moisture more readily. A material with good durability called PA6 is frequently used in climbing hands, automotive structural components, etc. Among the robust and durable materials utilized in industrial gears, such as marine propellers, is PA66. If a lightweight technical plastic with high impact and stress resistance is required, PA6 should be utilized^3^. Due to its shiny finish and simplicity in dyeing, it has a more aesthetically pleasing appearance than PA66. It is the best option for uses in the automotive, industrial, and military sectors^4,5^, which is the reason for the choice of the material in this study. Because of the excellent mechanical properties such as wear and abrasive resistance, superior strength, and toughness of PA6^6,7^, it is top ranked among the most employed chemical fibres in biomedical^8^, building and construction^9^, military, textiles industries, and in several other industries^10,11^. Coates et al.^12^ chemically recycled PA6 for polymer economy. Moustafa et al.^13^ reviewed and characterized PA6 as one of the ecofriendly and green polymeric materials. Li et al.^14^ mentioned that PA6 has a good electrochemical performance and can be applied for battery electrodes. These extensive applications of PA6 in several fields draw the attention of researchers in production and industrial finishing engineering to come up with better and optimal processing conditions for high-quality of PA6 during machine turning operation. Although, several studies have employed different optimization techniques in the literature, to the best of our knowledge, no study has been conducted in the employment of Probability multi-objective optimization technique to manufacture PA6 for industrial application.

Turning operation is one of the fundamental machining processes for the removal of some quantities of metal from a rotating workpiece through a single cutting tool moving parallel to the rotation axis^15,16^. The parameters of an employed machine such as cutting speed, depth of cut, tool geometry, feed rate, etc. are usually set to have as much as possible high quality of the manufactured material, because they are said to influence the quality output of the manufactured material^17–19^. To have a high-quality manufactured PA6, this paper employs a new and trending multi-criterial optimization tool (probability based multi-objective optimization tool). The machine parameters are conditioned through the help of the optimization tool for a better material removal rate (MRR) and for a low surface roughness. A multi-criterial optimization tool is chosen over a single optimization tool because of the two required performance characteristics (MRR and surface roughness) of PA6.

**Figure 1:**
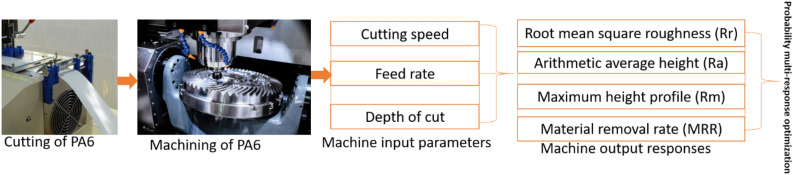
PA6 machining with probability multi-response optimization.

Numerous studies have been conducted in the use of multi-criterial decision-making tools for the optimization of machine parameters^20–22^. Abhishek et al.^6^ employed Taguchi technique to assist fuzzy linguistic reasoning multi-objective optimization of turning operation conditions for better performance of PA6. Mia et al.^23^ used grey relational multi-objective optimization analysis in MQL-assisted turning manufacturing. Warsi et al.^24^ employed a multi-objective optimization technique in the turning operation of aluminum-titanium alloy. Khan et al.^25^ employed a grey relational multi-objective optimization technique to turning operational conditions in order to manufacture high grade titanium-based alloy. Tanvir et al.^26^ employed an optimization algorithm to optimize multiple performance characteristics of stainless steel under turning operation. Gadagi and Adake^27^ employed a constrained multi-objective technique to optimize turning conditions through genetic algorithm and particle swarm optimization methods. Nguyen et al.^28^ employed an artificial neural network-based optimization method to optimize burning operation conditions for better surface characteristics of a workpiece. Fountas et al.^29^ employed orthogonal design of experiment with the assistance of neural network on the optimization of 5-axis machined sculpture surface finishing. Kechagias et al.^30^ employed the design of experiment to investigate turning operation of reinforced polymer composites. Their results revealed that the arithmetic mean roughness, the maximum peak to valley and the fractal dimension solely depend on the federate parameter, however, it was discovered that regression modeling only applies for the arithmetic mean roughness. Kechagias et al.^31^ in another study employed Taguchi design with the assistance of full factorial design to investigate the machinability performance during dry longitudinal turning of Ti-6Al-4V-ELI titanium alloy. The results showed that Taguchi design is suitable to analyze machinability issues of “difficult-to-cut” materials. This review shows a possible employment of multi-objective optimization methods for the optimization of machine parameters for better performance of specific material in different applications. Clearly, there is a gap in the employment of deeper multi-objective optimization analysis of machine conditions for the manufacturing of PA6 (please see Fig. 1 for elucidation). Hence, this study employed a new and trending multi-criterial method in turning operation manufacturing of PA6. The multi-criterial method is used as a multi-criterial decision-making method for proper manufacturing of PA6. MRR and surface roughness are the performance indicators of the manufactured PA6.

There are different and several multi-criterial decision-making analyses that have been employed in the literature. A few out of many are: Ashby’s technique^32–34^; Genetic algorithm method^35–39^; Grey relational analysis^40–47^; fuzzy optimization technique^6,48–51^; TOPSIS method^52–55^. Among these many multi-objective optimization methods, Intersection multi-objective probability method is a recent and trending method that has been proven to be effective, efficient, and simple^56–60^. Hence, this study employed the probability multi-objective optimization technique to optimize turning operation conditions for proper multi-criterial decision making in the manufacturing of high-quality PA6.

## Materials and methods

The data used was obtained from the study of Abhishek et al.^6^ with the following procedures: as working material, samples of nylon 6 (density: 1.3 g/cm^3^) bars with ϕ50 × 150 (cutting length: 50 mm) dimensions were employed. The machining procedure was performed using an Indolov SHRIRAM IK-20 single point high speed steel (HSS) tool. To improve the machining conditions in the experiment, three cutting parameters—speed, feed, and depth of cut—were changed at three distinct levels. The machining was done using a PINACHO manual lathe (180 × 750) from Tussor Machine Tool India Pvt. Ltd. in Coimbatore, India. Additionally, corresponding MRR values have been calculated. The Talysurf (Taylor Hobson, Surtronic 3 +) has a stylus that skids over the surface in accordance with the carrier modulating principle, allowing for the measurement of surface roughness. Experimental information (measured response parameters) is shown in Table 1. The DHD 200 Macro single pan DIGITAL reading electrically controlled analytical balance, manufactured by Dhona Instruments, was used to measure the weight of the work piece. The curated data in their study is presented in Table 1. While the optimization analysis method employed is shown in underneath:

**Table 1:** Experimental runs and responses.

Experimental runs	Experimental runs			Experimental responses			
	Cutting speed (rpm)	Feed rate (mm/rev)	Depth of cut (mm)	Rr	Ra	Rm	MRR
1	360	0.083	2	1.61333	1.35	8.43333	1436.84
2	360	0.166	3	5.01333	4.19	39.8	3992.67
3	360	0.331	4	5.56333	4.76	23.5333	9909.79
4	530	0.083	3	2.33333	1.78667	18.3967	4290.98
5	530	0.166	4	3.1	2.64	12.6333	7693.07
6	530	0.331	2	5.33667	4.65333	20.0667	5298.24
7	860	0.083	4	1.06667	0.858	6.82333	6048.7
8	860	0.166	2	3.47667	2.97667	13.3667	4762.78
9	860	0.331	3	4.69667	4.24333	18.0333	18,843.2

### Probability multi-objective optimization method

#### Beneficial utility index method

Beneficial utility index analysis was employed on the response whereby as much as possible is required. The larger is better is the requirement for the material removal rate characteristic, hence the beneficial utility index was employed for it. This means the index response has a linear and a positive contribution to the partial preferable probability. Equation (1)^56^ was employed to calculate the partial positive probability index (*Pij*). Equation (2)^56^ was employed to normalize the factor (α_*j*_) of *j*th utility index of beneficial response, material removal rate (MRR).

1 $$ Pij = \alpha_{ij} X_{ij} ,\quad i \, = 1,2, \ldots ,n;\,j = 1,2, \ldots m, $$

2 $$ \alpha_{j} = { 1}/({\text{n}} \bar{X}_{J}  ). $$

Note that *X*_*ij*_* is the* jth beneficial utility index of response of the ith number of samples, n is the considered number of experiments in the study, while m is the utility indices of each sample involved, ${X}_{j}$ is the arithmetic mean of the utility index of the response. The analyzed data for the MRR, P(MRR) is presented in Table 2.

**Table 2:** Probability analysis.

Experimental runs	Beneficial and unbeneficial probability analysis				Probability products and ranking	
	P(Rr)	P(Ra)	P(Rm)	P(MRR)	Pt × 10^4^	Rank
1	0.1826	0.1847	0.1477	0.0231	1.1498	6
2	0.0589	0.0618	0.0264	0.0641	0.0616	9
3	0.0388	0.0371	0.0893	0.1591	0.2049	7
4	0.1564	0.1658	0.1092	0.0689	1.9513	3
5	0.1285	0.1289	0.1315	0.1235	2.6902	2
6	0.0471	0.0418	0.1027	0.0851	0.1718	8
7	0.2025	0.206	0.154	0.0971	6.2391	1
8	0.1148	0.1143	0.1286	0.0765	1.2911	5
9	0.0704	0.0595	0.1106	0.3026	1.4012	4

#### Unbeneficial utility index method

The unbeneficial utility index analysis was employed on the as small as possible response is desired. The other three responses considered in the study are the root mean square roughness (Rr), arithmetic average height (Ra), and maximum height profile (Rm). These are surface roughness-based characteristics. As small as possible is desired for better surface finishing of PA6 is needed. The three index responses contribute negatively to the partial preferable probability. Equation (3)^56^ is used to calculate the partial negative probability index (*Pij*), while Eq. (4)^56^ was employed to normalize the response factor (*βj*) of the jth utility index. Table 2 presents the probability, P optimization analysis data. In Eqs. (3) and (4), X_jmax_ and X_jmin_ represent the maximum and minimum values of the experimental response indicator X_j_ in the response group, respectively.

To conclude the multi-objective optimization analysis, the four probability response data were singularized using by multiplying the individual partial preferable probability of each experimental run by one another, which is in accordance with the basic probability theory^56,61^, i.e., the total favorable probability of the ith candidate factor to be selected is the product of its partial favorable probability of each factor response indicator P_ij_ ("joint probability" or "intersection" in set theory). Next, the corresponding probability product was ranked based on the best response performance in ascending order. Table 2 presents the finalized probability product of each experimental run and its ranking.

3 $$ Pij = \beta_{ij} (X_{jmax} + X_{jmin} - \, X_{i} ),\quad i = 1, \, 2, \ldots ,n;j = 1,2, \ldots m, $$

4 $$ \beta ij = {1}/\left[ {n\left( {X_{{j{\text{min}}}} + X_{{j{\text{max}}}} } \right) - nX_{j} } \right]. $$

## Results and discussion

### Multi-criterial decision-making analysis

**Figure 2:**
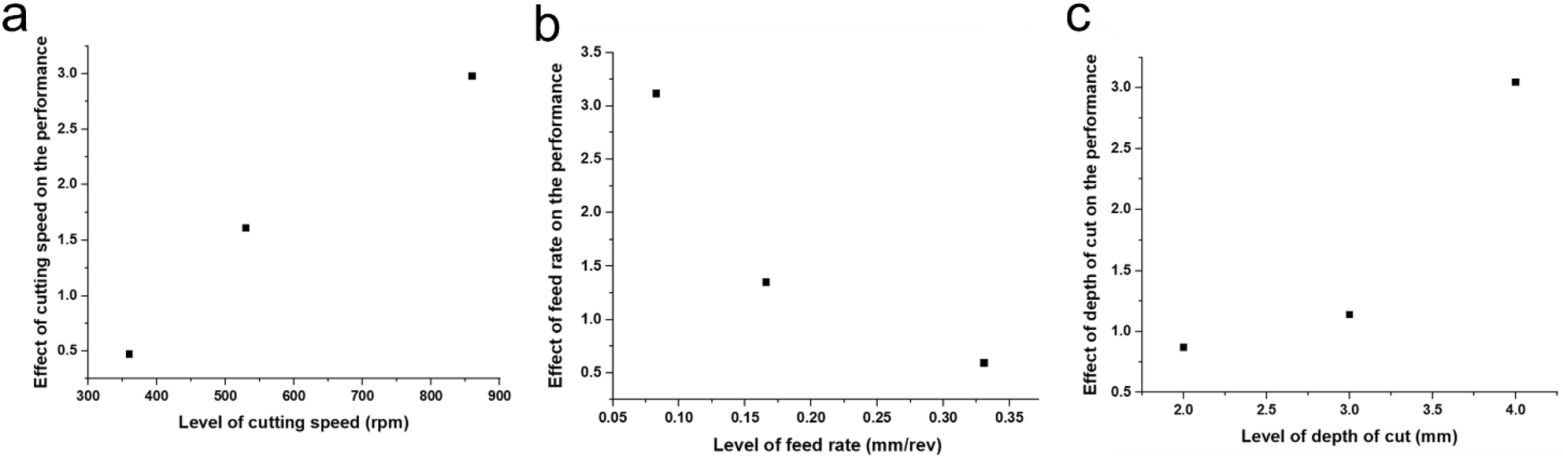
Effect of machine settings on Multi-performance characteristic.

Figure 2 presents the graphical representation of each turning operational conditions (machine settings) against the probability product (Pt) (multi-performance characteristic) of the experimental runs. This gives the effect and optimization of machine conditions on the Pt of the manufactured PA6. The presentation intends to assist in making multi-criterial decisions for production and research engineer. The graph reveals that an increase in cutting speed and depth of cut increased the Pt, i.e., they contribute positively to the multi-performance response of the manufactured PA6^62,63^. But it is shown that an increase in feed rate decreased the Pt of the manufactured PA6^64,65^. In other words, the results revealed that it imperative to take caution in the adjustment of feed rate of machine during the production of PA6.

Table 3 gives a numerical representation of each turning operational conditions against the probability product (Pt) of the experimental runs. From the table, the optimal turning operational conditions are obtained at Level 3 for the cutting speed and the depth of cut, while Level 2 is the optimal condition for the feed rate. This observation is also reflected in Fig. 2 and Table 2. Hence, the 7th experimental shows the optimal multi-performance characteristics, and it is ranked number one accordingly (Table 3).

**Table 3:** Optimal machine settings.

Factors	Settings
Cutting speed (rpm)	860
Feed rate (mm/rev)	0.083
Depth of cut (mm)	4

Table 4 presents the analysis of variance (ANOVA) of all the turning operational conditions on the manufactured PA6. This presentation substantially and quantitatively revealed the level of significance of the machine conditions as fairly presented in Table 5. It is shown that the feed rate which is the most significance condition has a percentage of contribution of 34.09%, while the cutting speed shows a percentage of contribution of 32.05%, then depth of cut having a contribution of 28.62%. It is expedient to state that the residual error is less significant on the overall Pt, having a contribution of 5.24%. The order of significance of the turning operational conditions are displayed by delta of the Pt. A higher delta value exhibits a higher significance, and this is ranked accordingly. Hence, the feed rate is the most significant condition, followed by cutting speed, and then depth of cut.

**Table 4:** Analysis of variance of turning operation conditions.

Factors	DOF	Adj SS	Adj MS	F	Contribution (%)	Remark
Cutting speed (rpm)	2	9.442	4.7208	6.12	32.05	Significant
Feed rate (mm/rev)	2	10.042	5.0211	6.51	34.09	Most significant
Depth of cut (mm)	2	8.432	4.2162	5.47	28.62	Significant
Residual error	2	1.543	0.7714		5.24	Less significant
Total	8	29.459	14.7295	S = 0.8783	R^2^ = 94.8%	R^2^_Adj_ = 79.1%

**Table 5:** Numerical representation of the turning operation.

Factors	Cutting speed	Feed rate	Depth of cut
Level 1	0.4721	3.1134	0.8709
Level 2	1.6044	1.3476	1.1380
Level 3	2.9771	0.5927	3.0447
Delta	2.5050	2.5207	2.1738
Rank	2	1	3

The value of R^2^ and R^2^_adj_ reflect the model fitness of the experimental design, and their high and close values showed that the model has a good fitness. These findings showed a less worries in the making of the material handling multi-criterial decision for high-quality of PA6.

### Confirmation analysis

Sequel to the determination of the optimal turning operational conditions on the multi-response, Pt of the manufactured PA6, it is important to compute their corresponding predicted results and compare them with their experimental counterparts. Equation (5) was employed to compute the predicted optimal responses and GRG^41,66^. The predicted value as computed based on Eq. (5) is 5.766. This shows a close value with the experimental value, as displayed in the 7th experimental run, which is 6.2391. For a very clear confirmation, Eq. (6) was employed to determine the Confidence interval (CI) and it is used to check the closeness of the experimental responses as compared to the predicted responses^41,67^. Hence, the computed confidence interval value is gotten to be 5.0383. The 95% confidence level of the predicted optimal response is displayed in Eq. (8), as highlighted by Abifarin^41^. The actual boundary of the optimization analysis based on Eq. (8) is shown in Eq. (9). This shows that the multi-objective optimization analysis is within the 95% confidence level. It is important to say that the confidence bound gives room for many probable experimental values, in the case that a different type of machine is employed.

Where; ${\gamma }_{m}$ is the average mean of each response for the whole design runs, ${\gamma }_{0}$ is the highest value of response at different optimal turning operational conditions and q is the number of the conditions. ${F}_{\propto }(1,{f}_{e})$ is the F ratio for $\alpha$ (risk); ${f}_{e}$ = degree of freedom (DOF) of residual error; V_e_ is the variance error; ${\eta }_{eff}$ is the adequate number of repetitions as computed in Eq. (7). R shows the total number of repetitions for confirmation; N is the total number of experiments.

5 $${\gamma }_{predicted}={\gamma }_{m}+\sum_{i=1}^{q}{\gamma }_{0}-{\gamma }_{m},$$

6 $$CI=\sqrt{{F}_{\propto }(1,{f}_{e}){V}_{e}\left[\frac{1}{{\eta }_{eff}}+\frac{1}{R}\right]},$$

7 $${\eta }_{eff}=\frac{N}{1+(total\,DOF\,of\,control\,factors)},$$

8 $${\gamma }_{predicted}-CI<{\gamma }_{experimental}<{\gamma }_{predicted}+CI,$$

9 $$ 0.7277<{\gamma }_{experimental}<10.8043.$$

### Managerial implications

The work mentioned above optimizes the quality and productivity of a manufacturing process. In order to reduce machining costs and production times, boost productivity, and improve product quality, manufacturing processes frequently need to optimize their machining parameters. The current work, which uses Taguchi's resilient design technique in conjunction with Probability multi-response optimization reasoning, illustrates a multi-response optimization problem for the selection of optimal cutting parameters (optimal process environment) for turning (machining) of nylon 6 as a case study. Cutting speed, feed, and depth of cut have all been taken into consideration in this study in order to achieve the desired MRR of the process and favorable multiple surface roughness features for the machined product; based on L9 orthogonal array experimental design. The goal of the study is to find a suitable process environment for the beneficial simultaneous optimization of quality and productivity. Numerous statistically significant surface roughness measures (of the machined product) have been regarded as product quality attributes, whereas MRR has been used as a productivity indicator for the aforementioned machining process. Use of a Probability multi-response optimization technique has been suggested for meaningful and practical aggregation of individual performance indices into an equivalent single quality index, thereby converting such a multi-objective optimization problem into an equivalent single objective optimization. This avoids assumptions, limitations, uncertainties, and imprecision in application of existing multi-response optimization techniques (like Taguchi-Grey, desirability function based Taguchi, utility concept based Taguchi, Fuzzy inference system, etc.). Using the Taguchi approach, multi-performance characteristics measured have finally been optimized. The study shows that the proposed approach may be applied successfully, and the results of the confirmatory test were satisfactory. The suggested method can be applied successfully to any multi-response optimization issue in an off-line quality control setting.

## Conclusions

Despite complications encountered in the multi-performance of machining on the production of PA6, this study has successfully applied probability multi-objective optimization method in the multi-criterial determination of optimal turning operational conditions of a manufacture PA6. The results revealed that the optimal conditions for the ones with the 7th experimental run, which are 860 rpm cutting speed, 0.083 mm/rev feed rate, and 4 mm depth of cut. Furthermore, the analysis of variance and the numerical presentation of the turning operational conditions revealed that feed rate is the most significant condition with a contribution of 34.09%, followed by cutting speed with a contribution of 32.05%, and then depth of cut with a contribution of 28.62%. Also, the confirmation analysis revealed a very high efficacy of the multi-objective optimization method employed in this study. This suggests that probability based multi-objective optimization is efficacious for optimizing machine conditions of any manufactured engineering material. It is interesting to state that the high confidence level placed on the considered turning operational conditions gives room for probable machine conditions adjustments for better PA6 in the case where different machine types are employed.

This study recommends the optimal settings derived from this study for the production of PA6 under machine conditions. This recommendation addresses multi-performance optimization complications that may be encountered by researchers and production engineers in the field of machine and material manufacturing. Although, probability multi-objective optimization technique has been proven to be effective in several applications, this study recommends further study on the comparison between the other optimization techniques and probability multi-objective optimization technique.

## Data Availability

All data generated or analyzed during this study are included in this published article (Table 1 in the manuscript presents the data analyzed).
